# Association between a *C8orf13–BLK* Polymorphism and Polymyositis/Dermatomyositis in the Japanese Population: An Additive Effect with *STAT4* on Disease Susceptibility

**DOI:** 10.1371/journal.pone.0090019

**Published:** 2014-03-14

**Authors:** Tomoko Sugiura, Yasushi Kawaguchi, Kanako Goto, Yukiko Hayashi, Takahisa Gono, Takefumi Furuya, Ichizo Nishino, Hisashi Yamanaka

**Affiliations:** 1 Institute of Rheumatology, Tokyo Women's Medical University, Tokyo, Japan; 2 Department of Neuromuscular Research, National Institute of Neuroscience, and Department of Clinical Development, Translational Medical Center, National Center of Neurology and Psychiatry, Tokyo, Japan; Friedrich-Alexander University Erlangen, Germany

## Abstract

**Background:**

Accumulating evidence has shown that several non-HLA genes are involved in the susceptibility to polymyositis/dermatomyositis. This study aimed to investigate the involvement of *C8orf13–BLK*, one of the strongest candidate genes for autoimmune diseases, in susceptibility to polymyositis/dermatomyositis in the Japanese population. A possible gene–gene interaction between *C8orf13–BLK* and *STAT4*, which we recently showed to be associated with Japanese polymyositis/dermatomyositis, was also analyzed.

**Methods:**

A single-nucleotide polymorphism in *C8orf13–BLK* (dbSNP ID: rs13277113) was investigated in the Japanese population using a TaqMan assay in 283 polymyositis patients, 194 dermatomyositis patients, and 656 control subjects.

**Results:**

The *C8orf13–BLK* rs13277113A allele was associated with overall polymyositis/dermatomyositis (*P*<0.001, odds ratio [OR] 1.44, 95% confidence interval [CI] 1.19–1.73), as well as polymyositis (*P* = 0.011, OR 1.32, 95% CI 1.06–1.64) and dermatomyositis (*P*<0.001, OR 1.64, 95% CI 1.26–2.12). No association was observed between the *C8orf13–BLK* rs13277113A allele and either interstitial lung disease or anti-Jo-1 antibody positivity. The *C8orf13–BLK* rs13277113 A and *STAT4* rs7574865 T alleles had an additive effect on polymyositis/dermatomyositis susceptibility. The strongest association was observed in dermatomyositis, with an OR of 3.07 (95% CI; 1.57–6.02) for the carriers of four risk alleles at the two SNP sites, namely, rs1327713 and rs7574865.

**Conclusions:**

This study established *C8orf13–BLK* as a new genetic susceptibility factor for polymyositis/dermatomyositis. Both *C8orf13–BLK* and *STAT4* exert additive effects on disease susceptibility. These observations suggested that *C8orf13–BLK*, in combination with *STAT4*, plays a pivotal role in creating genetic susceptibility to polymyositis/dermatomyositis in Japanese individuals.

## Introduction

Polymyositis and dermatomyositis are rare connective tissue diseases, with unknown etiologies, which belong to the idiopathic inflammatory myopathies (IIMs). The typical clinical features are symmetrical and include proximal weakness of skeletal muscles and infiltrating mononuclear cells seen in muscle biopsies, and may be accompanied by skin rash. The diagnosis of IIMs in 29% of patients is accompanied by other connective tissue diseases (CTDs), such as systemic sclerosis (SSc), systemic lupus erythematosus (SLE), and rheumatoid arthritis (RA) [1], suggesting that IIMs are associated with general autoimmunity.

Although most immunogenetic IIM investigators have focused on the polymorphic genes of the major histocompatibility complex (human leucocyte antigen [HLA]) [2], new genetic markers have been identified outside the HLA region. For example, the R620W polymorphism of the protein tyrosine phosphatase N22 gene (*PTPN22*), one of the most well-documented risk genes for several autoimmune diseases specific for Caucasians [3], was found to be associated with IIMs in British Caucasian patients [4]. Moreover, we have recently shown that a polymorphism (rs7574865) in the signal transducer and activator of transcription 4 gene (*STAT4*) is associated with adult-onset polymyositis and dermatomyositis in a Japanese population [5]. After being identified as a risk gene for SLE and RA [6], *STAT4* was also associated with susceptibility to a number of other autoimmune diseases, irrespective of ethnicity [7]. These observations strongly suggested that IIMs share an ‘autoimmune-prone’ genetic background with other autoimmune diseases.

*BLK* encodes a B lymphoid-specific tyrosine kinase of the Src family, which is involved in B cell receptor-mediated signaling and B cell development [8]. The risk allele (A) of rs13277113 (rs13277113A) within the *C8orf13–BLK* region of chromosome 8p23–p22 was originally identified in SLE patients by a genome-wide association study (GWAS) [9]. This polymorphism is associated with low levels of *BLK* mRNA and high levels of *C8orf13* mRNA, which encodes a ubiquitously expressed gene of unknown function [9]. An association between *C8orf13–BLK* polymorphisms and SLE was first identified in North Americans of European descent and in Swedish populations [9], and was later replicated in both European [10] and Asian populations [11]. Subsequently, other autoimmune diseases, such as SSc [12], [13] and RA [14], were shown to be associated with polymorphisms in *C8orf13–BLK*.

The contribution of *C8orf13–BLK* appears to be prominent in Asian populations, in which the risk allele rs13277113A is the major allele. Indeed, the allele frequency of rs13277113A is approximately 0.65 in the Japanese population [11], [12], compared with approximately 0.25 in North American and European populations [9], [13], [14]. In Japanese SLE patients, a positive association between disease susceptibility and this polymorphism in *C8orf13–BLK* was confirmed with an OR of 2.44 [11], whereas the OR was 1.39 in Caucasian populations [9]. A similar increase in OR was observed in Japanese SSc patients compared with Caucasian patients [12], [13].

Therefore, genetic variants of *C8orf13–BLK* could strongly contribute to lowering the disease threshold for autoimmune diseases, and particularly in Asian populations. In this study, we investigated whether *C8orf13–BLK* variants contribute to disease susceptibility in Japanese polymyositis/dermatomyositis patients and assessed any potential additive effects between *C8orf13–BLK* and *STAT4* in the susceptibility to polymyositis/dermatomyositis.

## Patients and Methods

### Subjects

This study was reviewed and approved by the research ethics committees of both the Tokyo Women's Medical University (TWMU) and National Center of Neurology and Psychiatry (NCNP) and complied with the Helsinki Declaration.

We enrolled patients who had probable or definite myositis based on the criteria of Bohan and Peter [15] and who were 18 years of age or older at disease onset. For our study, dermatomyositis patients included those with clinically defined amyopathic dermatomyositis who fulfilled the traditional criteria of Sontheimer [16]. Patients with myositis overlapping with other CTDs, such as RA, SLE, and SSc, were excluded from the study because these CTDs have previously been associated with *C8orf13–BLK* variants [9]–[14]. Patients with inherited, metabolic, or infectious myopathies and with inclusion body myositis were also excluded. All patients underwent a muscle biopsy.

The polymyositis/dermatomyositis patients were recruited from two different institutions: 138 (46 polymyositis and 92 dermatomyositis patients) were recruited from the Institute of Rheumatology, TWMU (Tokyo, Japan), and 339 (237 polymyositis and 102 dermatomyositis patients) were recruited from the National Institute of Neuroscience, NCNP (Kodaira City, Tokyo, Japan). In total, 477 patients with adult-onset polymyositis/dermatomyositis (69.8% female) were retrospectively investigated, including 283 polymyositis patients (68.3% female) and 194 dermatomyositis patients (71.1% female). The mean ages of the polymyositis and dermatomyositis patients were 51.4±15.8 and 52.3±16.5 y, respectively. None of the patients were genetically related.

As controls, we enrolled healthy unrelated individuals from the Tokyo area (n = 656; 57.1% female; mean age = 38.6±11.9 years). All patients and control subjects were Japanese individuals, and they were living in the central part of mainland Japan (Honshu).

For a sub-analysis of association between the *C8orf13–BLK* rs13277113 polymorphism and the presence or absence of interstitial lung disease (ILD) or serological status, 138 polymyositis/dermatomyositis patients recruited from TWMU were evaluated. The presence of ILD was confirmed or excluded by computed tomography (CT), high-resolution CT, if available, and spirometry. For serological analysis, the only association between the possession of the anti-Jo-1 antibody and *C8orf13–BLK* rs13277113A was analyzed, because not all patients were screened for other myositis-specific autoantibodies (MSAs).

### Genotyping

To date, rs13277113 within *C8orf13–BLK* and the related single nucleotide polymorphism (SNP) have shown the strongest association with several autoimmune diseases [9]–[14]. Given this background, and our previous findings, the *C8orf13–BLK* rs13277113 and *STAT4* rs7574865 genotypes were determined using a TaqMan fluorogenic 5′-nuclease assay, according to the manufacturer's instructions (Applied Biosystems, Carlsbad, CA, USA). End-point fluorescence was measured with an ABI Prism 7900 HT Sequence Detection System (Applied Biosystems). In the disease subgroups and the control group, none of the SNPs deviated from Hardy–Weinberg equilibrium.

### Statistical analysis

Association analyses were performed using chi-square tests for 2×2 contingency tables. Bonferroni's correction was applied for association analyses between the *C8orf13–BLK* polymorphism and the three clinical subsets (polymyositis, dermatomyositis, and polymyositis/dermatomyositis patients versus controls) and was expressed as *Pcorr*. The odds ratios (ORs) and 95% confidence intervals (95% CIs) were also determined. A logistic regression model was applied to assess gene–gene interactions between *C8orf13–BLK* rs13277113 and *STAT4* rs7574865 by using SPSS (Statistical Package for the Social Sciences) software version 19.0 (SPSS, Chicago, IL, USA) and to determine the additive effects of these two SNPs. Regression analysis accounted for the combination of the genotypes from both loci; thus, each individual had 0–4 risk alleles when considering both SNP sites. The ORs were computed using a logistic regression model, with individuals carrying 0 or 1 risk allele as a reference. The difference in the *C8orf13–BLK* and *STAT4* risk allele counts between the patients and control subjects was analyzed using Fisher's exact test. Statistical analyses were conducted using SPSS version 19.0 (SPSS).

Power calculations were performed using the Quanto software (http://hydra.usc.edu/gxe/) for case–control analysis, using a significance level of 0.05. Power was calculated to be 0.78 using the ORs previously reported in Japanese collagen disease [12] and the present study, as well as the sample size and risk allele frequency in the present study. Under the same parameter settings, 503 patients would be needed to demonstrate an OR of 1.44, at an alpha of 0.05, with power of 0.8. Similarly, to gain power of 0.9, 665 patients would be needed.

## Results

### Association of *C8orf13–BLK* rs13277113 with polymyositis/dermatomyositis in the Japanese population

The frequency of the *C8orf13–BLK* rs13277113 A allele was in good agreement with those previously reported for the Japanese population [11], [12]. In the present study, the A (risk) allele of rs13277113 was found in 72% of the chromosomes in the polymyositis patients, 76% in the dermatomyositis patients, and 74% in the polymyositis/dermatomyositis patients. All frequencies in the disease subsets were significantly higher than those in the control subjects (64%; *Pcorr* = 0.033, OR 1.32 for polymyositis; *Pcorr* = 4.5×10^−4^, OR 1.64 for dermatomyositis; and *Pcorr* = 3.3×10^−4^, OR 1.44 for polymyositis/dermatomyositis). Comparisons of the genotypes showed association of the rs13277113 A allele in a dominant model with dermatomyositis (rs13277113 A/A or A/G genotype; *Pcorr* = 0.0011, OR 4.73). The allele and genotype frequencies are detailed in Table 1.

**Table 1:** Association between *C8orf13–BLK* rs13277113 and polymyositis/dermatomyositis.

Subjects (n)	PM (283)	DM (194)	PM+DM (477)	controls (656)
A allele (frequency)	407 (0.72)	295 (0.76)	702 (0.74)	865 (0.65)
allelic association				
OR (95%CI)	1.32 (1.06–1.64)	1.64 (1.26–2.12)	1.44 (1.19–1.72)	Referent
*P*	0.011	1.5×10^−4^	1.1×10^−4^	-
Corrected *P*	0.033	4.5×10^−4^	3.3×10^−4^	-
A/A+A/G (frequency)	262 (0.92)	189 (0.97)	451 (0.94)	583 (0.89)
genotype association				
OR (95%CI)	1.56 (0.94–2.59)	4.73 (1.88–11.9)	2.17 (1.37–3.46)	Referent
*P*	N.S.	3.6×10^−4^	8.8×10^−4^	-
Corrected *P*	N.S.	0.0011	0.0026	-

OR: Odds ratio, CI: confidence interval, PM: polymyositis, DM: dermatomyositis, N.S.: not significant.

In the sub-analysis of 138 patients, comprising 46 with polymyositis and 92 with dermatomyositis, the complication of ILD was observed in 46.5% of the polymyositis patients and 66.3% of the dermatomyositis patients; in the combined cohort, 59.8% had ILD. The rs13277113A frequency was equal between patients with ILD (0.75) and those without (0.75). Of the 138 polymyositis/dermatomyositis patients recruited from TWMU, 20.4% were positive for the anti-Jo-1 antibody. The rs13277113A frequency was not statistically significantly different between anti-Jo-1 antibody-positive and antibody-negative patients (0.73 vs. 0.75, respectively). Therefore, no association was found between the rs13277113 polymorphism and the ILD disease phenotype or anti-Jo-1 antibody positivity.

### Additive effects of *C8orf13–BLK* and *STAT4*

An additive effect of both risk alleles (the *C8orf13–BLK* rs13277113A allele and the *STAT4* rs7574865 T allele) on susceptibility to polymyositis, dermatomyositis, and polymyositis/dermatomyositis was observed (Table 2).

**Table 2:** A cumulative effect of risk allele number (*C8orf13–BLK* rs13277113A and *STAT4* rs7574865T) on susceptibility to polymyositis, dermatomyositis, and polymyositis/dermatomyositis.

No. of risk alleles	PM (283)		DM (194)		PM+DM (477)	
	OR (95%CI)	*P*	OR (95%CI)	*P*	OR (95%CI)	*P*
0+1	Referent	-	Referent	-	Referent	-
2	1.12 (0.78–1.62)	N.S.	1.71 (1.09–2.57)	0.017	1.34 (0.98–1.83)	N.S.
3	1.37 (0.91–2.03)	N.S.	2.18 (1.36–3.48)	1.8×10^−3^	1.64 (1.17–2.29)	3.8×10^−3^
4	2.47 (1.40–4.35)	1.7×10^−3^	3.07 (1.57–6.02)	1.1×10^−3^	2.67 (1.61–4.42)	1.4×10^−4^

OR: Odds ratio, CI: confidence interval, PM: polymyositis, DM: dermatomyositis, N.S.: not significant.

The OR for polymyositis patients carrying four risk alleles was 2.47 (95% CI 1.40–4.35), using individuals with 0 or 1 allele as a reference. The ORs for dermatomyositis gradually increased: 1.71 (95% CI 1.09–2.57) for carriers of two risk alleles, 2.18 (95% CI 1.36–3.48) for carriers of three risk alleles, and 3.07 (95% CI 1.57–6.02) for carriers of four risk alleles. The ORs for the polymyositis/dermatomyositis patients also gradually increased: 1.64 (95% CI 1.17–2.29) for carriers of three risk alleles and 2.67 (95% CI 1.61–4.42) for carriers of four risk alleles. Therefore, additive effects of *C8orf13–BLK* and *STAT4* were observed, most notably in dermatomyositis.

## Discussion

IIMs are clinically and serologically heterogeneous disorders. To date, the genetic basis of IIMs appears to involve at least two major components, viz., HLA regions and non-HLA risk genes common to other autoimmune diseases. The HLA region is associated with overall IIMs susceptibility particularly in Caucasians, in whom the HLA8.1 ancestral haplotype containing DRB1*0301 allele is prevalent, and is tightly linked to production of myositis-specific autoantibodies (MSAs) [2]. However, the association between the HLA region and IIMs is lost in Mexican-American and Korean populations [17]. In the Japanese population, in which the DRB1*0301 allele is rare (0.1–0.2% of the population), DRB1*0803 is weakly associated with susceptibility to IIMs and carriage of anti-aminoacyl-tRNA synthetases (ARS) autoantibodies [18]. Therefore, it seems to be likely that the HLA region is associated with IIM susceptibility to different degrees in different ethnicities, and that it is tightly associated with MSA production. On the other hand, non-HLA risk genes that encode the immune response or cell signaling regulatory proteins are involved in the susceptibility to IIMs, regardless of the presence or not of MSA [2], [4], [5], [19]. Since such risk genes outside of the HLA region are common to other autoimmune diseases, IIMs are likely to share genetic etiology with other autoimmune diseases.

This study presents an association between polymyositis/dermatomyositis and *C8orf13–BLK* rs13277113A in the Japanese population. While preparing this manuscript, data of a GWAS on dermatomyositis in adults and juveniles of European ancestry (n = 1178) were published [19]. According to that study, *BLK* rs2736340 was identified as one of the risk genes for adult and juvenile dermatomyositis in Europeans after screening of 141 non-MHC SNPs that had previously been associated with autoimmune diseases [19]. Because both *BLK* rs2736340 and rs13277113, which were investigated in the present study, are in complete linkage disequilibrium, the risk haplotype identified by GWAS and by the present study are identical. The present Japanese case–control study, as a result, replicated the study of the European GWAS study. To date, few susceptibility genes for IIMs have been replicated, except for the HLA 8.1 haplotype in Caucasians, probably due to the different risk allele frequencies in different ethnicities, relatively low disease prevalence, and disease heterogeneity. The present data highlighted the strong contribution of *BLK* to polymyositis/dermatomyositis susceptibility, irrespective of ethnicity.

Accumulating evidence has shown that *BLK* is strongly involved in the development of a wide variety of autoimmune diseases [9]–[14]. However, it remains unclear how an autoimmune-risk variant within *C8orf13–BLK* influences Blk protein expression, results in altered B cell signaling. Although a risk variant in *C8orf13–BLK* reduces *BLK* mRNA transcript expression in a B cell lymphoblastoid cell line [9], it is unclear whether the variant affects protein expression. However, a recent report showed that the risk variant reduced Blk protein expression in B cells obtained from umbilical cord blood, although not in adult B cell subsets [20]. Reduced Blk expression in the early stage of B cell development may influence B cell receptor signaling, resulting in selection of autoimmune-prone B cells. *Blk*-knockout mice as well as *Blk* ^+/−^ mice exhibited an autoimmune phenotype, with a high titer of anti-nuclear antibody production compared with wild-type mice [21]. B cells are strongly involved in the humoral immune response, particularly as it pertains to autoantibody production.

Therefore, the idea that a risk allele of *C8orf13–BLK* is associated with autoantibody production seems to be reasonable. In the present sub-analysis, however, no increase was observed in the frequency of rs13277113A allele carriers in the anti-Jo-1 antibody-positive group of patients. Interestingly, similar results were previously obtained in SLE patients in whom *BLK* risk loci were not found to be associated with anti-DNA antibody production, although this gene increased disease susceptibility overall [22]. In human CD4^+^ cells, SNP-associated regulation of *BLK* expression has been found [23]. Therefore, although the mechanism underlying the triggering of autoimmune diseases by a *C8orf13–BLK* risk variant remains unknown, it may influence the overall immune response, including auto-reactive B cell selection or T cell function, resulting in altered individual immune response.

We have previously reported *STAT4* rs7574865 is associated with susceptibility to polymyositis/dermatomyositis in Japanese [5]. STAT-4 is a transcription factor that transduces IL-12, IL-23-, and type-1 interferon-mediated signals into Th1 and Th17 differentiation, monocyte activation, and interferon-gamma production [24]. Among many autoimmune disease-related genes, *STAT4* [25], [26], *C8orf13–BLK* [11], [12], as well as interferon regulatory factor 5 (*IRF5*) [27] seem to be the most representative susceptibility genes in the Japanese population. In particular, the genetic contribution of *C8orf13–BLK* [11], and to a lesser extent, of *STAT4* [25], are greater in the Japanese population compared with the Caucasian population, due to the high prevalence of the risk gene. Although each risk gene has a relatively low OR for disease susceptibility, the carriage of more risk alleles, in several risk genes, appears to increase the risk for disease susceptibility. Such cumulative associations have been shown in other autoimmune diseases [28], and now also here, by the discovery of the additive effect of alleles in *C8orf13–BLK* and *STAT4* in increasing the risk for polymyositis/dermatomyositis.

The major limitation of the present study was the paucity of association studies in clinical subsets, including serological phenotypes. However, despite the rarity of these diseases, we obtained a large sample size, which provided sufficient statistical power for this case–control study. We identified a susceptibility gene, *C8orf13–BLK*, for polymyositis/dermatomyositis. Both *C8orf13–BLK* and *STAT4* additively increased polymyositis/dermatomyositis susceptibility in the Japanese population.

### Key messages

- The *C8orf13–BLK* rs13277113A allele is associated with Japanese polymyositis/dermatomyositis.
- *C8orf13–BLK* rs13277113A and *STAT4* rs7574865T exert additive effects in polymyositis/dermatomyositis susceptibility.
